# Self-Directed Learning in Health Professions Education: A Systematic Review and Meta-Analysis

**DOI:** 10.5334/pme.2128

**Published:** 2026-01-28

**Authors:** Sean Wilkes, Lauren A. Maggio, Paolo C. Martin, John Melton, Binbin Zheng

**Affiliations:** 1 Uniformed Services University of the Health Sciences, Bethesda, Maryland, USA; 2 University of Illinois Chicago, College of Medicine, Chicago, Illinois, USA

## Abstract

**Introduction:**

This systematic review and meta-analysis evaluates the effectiveness of self-directed learning (SDL) in health professions education (HPE), examining its impact on knowledge, clinical performance, and behavioral outcomes. It investigates whether core SDL components influence educational outcomes, updating and extending the foundational work of Murad et al. (2010).

**Methods:**

We searched CINAHL, Embase, OVID Medline, PsycINFO, and Web of Science (2009–2023) for comparative studies evaluating SDL interventions in HPE. From 6,786 screened articles, 125 studies met inclusion criteria, with 48 eligible for meta-analysis. We conducted a three-level random-effects meta-analysis and moderator analyses on profession, outcome type, SDL modality, and facilitator role. Five independent reviewers conducted screening and extraction, resolving discrepancies via consensus.

**Results:**

The meta-analysis incorporated 74 effect sizes from 48 studies, revealing a small-to-moderate overall effect (Cohen’s d = 0.34, 95% CI 0.04, 0.64) with significant heterogeneity (I^2^ = 87%). SDL as intervention showed larger effects (d = 0.54 vs. d = –0.27, p = 0.004). Most studies involved Kirkpatrick Level 2 outcomes (knowledge/skills, 78%), with some Level 3 outcomes (skills/behaviors, 22%) and no Level 4 outcomes (patient/system) reported. Most teachers were absent or acted as facilitators, while learners were less likely to be involved in choosing resources (21%) or in assessments (25%).

**Conclusions:**

This updated meta-analysis reaffirms that SDL reliably enhances knowledge acquisition but suggests that it may yield only modest gains in clinical skills and behaviors. The wide variability in how SDL is defined and reported underscores the need for a consensus definition of SDL.

## Introduction

In health professions education (HPE), self-directed learning (SDL) is recognized as an effective instructional strategy that aligns with the core principles of adult learning [1]. Rather than passively absorbing information, learners in SDL take ownership of their educational process—defining objectives, selecting resources, and engaging in self-assessment. Such autonomy not only promotes intrinsic motivation, self-efficacy, and academic performance, but also fosters critical skills such as reflection, inquiry, and adaptability, which are essential for lifelong learning and professional development [2,3]. Aligning with Adult Learning Theory, Knowles [4] defines SDL as “a process in which individuals take the initiative, with or without the help of others, in diagnosing their learning needs, formulating learning goals, identifying resources for learning, choosing and implementing appropriate learning strategies, and evaluating learning outcomes.” Knowles further delineates the essential components of SDL: (1) educators serve as facilitators of learning rather than sources of content; (2) learners are involved in determining learning needs, objectives, and resources; and (3) learners are involved in directing the implementation and evaluation of the learning process [4].

In recent years, institutional frameworks have further underscored SDL’s importance. The Accreditation Council for Graduate Medical Education (ACGME), for example, identified SDL as a key component of the core competency of practice-based learning and improvement (PBLI), requiring residents to identify learning needs, engage in continuous self-assessment, and apply new knowledge to improve care [5]. Similarly, the American Medical Association’s Master Adaptive Learner model reinforces SDL as an essential attribute for developing capable and resilient physicians to continuously learn, unlearn, and relearn in response to changing clinical demands and contexts [6].

Knowles’ andragogy is often cited as the foundational framework for SDL, particularly in adult education and HPE, because it emphasizes autonomy, self-assessment, and learner-driven goal-setting [7]. While empirical evidence for andragogy itself is limited and its assumptions have been critiqued (e.g., that all adult learners inherently seek autonomy) [8], it remains a central touchstone for conceptualizing SDL in HPE [1]. At the same time, SDL cannot be understood solely through andragogy. Cognitive load theory suggests that SDL effectiveness depends on learners’ prior knowledge and the complexity of tasks [9]. Self-determination theory emphasizes the role of autonomy, competence, and relatedness in fostering intrinsic motivation essential for SDL [10,11]. Additionally, self-regulated learning (SRL) frameworks from educational psychology provide empirical models for understanding metacognitive processes in SDL [12].

While SDL and SRL share conceptual overlap, they emerge from different theoretical traditions. SDL, rooted in adult education, is often described as a pedagogical approach that foregrounds learner autonomy across the entire learning process, including diagnosing learning needs, selecting resources, and evaluating outcomes. In contrast, SRL, developed within educational psychology, emphasizes the metacognitive, motivational, and behavioral strategies learners use to regulate their own learning [13,14]. Prior reviews in HPE [15,16] have examined SDL extensively. Brydges et al. (2015) highlighted how SRL processes played a critical role in simulation-based education, emphasizing the importance of structured support to help learners regulate their practice. Van Houten-Schat et al. (2018) provided a broader synthesis across HPE contexts, underscoring how contextual factors—such as supervision, assessment demands, and institutional culture—influence learners’ ability to effectively self-regulate. For this review, we intentionally delimit our scope to studies explicitly framing interventions as SDL to ensure conceptual coherence and avoid conflating constructs. At the same time, we acknowledge that SRL literature offers valuable complementary insights, and that continued integration of SDL and SRL perspectives is essential for advancing educational design and research in HPE.

Despite general acceptance of SDL’s theoretical underpinnings, its implementation varies widely [17]. Some educational programs emphasize SDL’s essential components [18,19]—teachers as facilitators rather than content deliverers, learner participation in choosing learning resources and strategies, and the active self-assessment of achievements—while others adopt the SDL label without fully embracing these principles [20,21]. Moreover, SDL interventions can range from straightforward, computer-based modules to more complex curricular reforms that allow learners to navigate their own educational paths [22]. This variability in approach raises important questions: Which elements of SDL are most effective for improving knowledge and skills? Do certain learner groups or clinical training contexts offer more benefits than others? Answering these questions can help guide educators and decision-makers as they design and refine their curricula.

A systematic review published in 2010 [1] made a foundational step in synthesizing evidence on the effectiveness of SDL, showing at that time that SDL was associated with moderate knowledge gains and that it could be as effective as traditional methods in enhancing outcomes. However, the landscape of HPE has evolved significantly over the past decade, with increased emphasis on technological innovations, competency-based frameworks, and an intensified focus on adaptability. Complementing Murad’s findings, a subsequent systematic review by Cadorin et al. in 2017 focused on instruments used to evaluate SDL abilities among nursing students and professionals [23]. Their review highlighted both the increasing interest in measuring SDL as an educational outcome and the variability in how SDL is conceptualized and assessed across contexts. Importantly, they found that the operationalization of SDL remained inconsistent, reflecting ongoing challenges in defining and evaluating SDL competencies reliably. Building on these prior reviews, the current study advances the field by (1) examining SDL evolution in the era of competency-based and technology-enhanced education; (2) identifying the gap between SDL theory and practical implementation; (3) quantifying which SDL components are actually enacted in HPE contexts; and (4) providing evidence-based guidance for how educators can implement SDL effectively.

## Methods

This is a systematic review and meta-analysis of SDL building upon previously established methodology in the 2010 paper by Murad et al. [1]. We aimed to identify and synthesize studies that evaluate how SDL interventions impact learner outcomes in HPE.

This systematic review was conducted and reported in accordance with PRISMA 2020 guidelines (Page et al., 2021) to ensure methodological rigor and transparency. We followed the PRISMA checklist for all applicable items, including comprehensive database searching, duplicate screening, systematic data extraction, and transparent reporting of results. While the review protocol was not pre-registered, which we acknowledge as a limitation, all other PRISMA recommendations were adhered to, including the use of a flow diagram to document study selection and the assessment of risk of bias through our quality appraisal process.

### Search Strategy

We searched the following databases: CINAHL, Embase, OVID Medline, PsycINFO, and Web of Science from August 12, 2009 to October 11, 2023. As an update, we limited our search start date to align with Murad’s final search date.

The research team collaboratively designed the database searches in consultation with an experienced medical librarian. The librarian conducted the searches on October 11, 2023. We included search terms related to SDL and HPE that combined controlled vocabulary and key terms optimized for each database. The search built on the earlier search strategy by Murad et al. (See Appendix A for complete search strategies). We limited our search to English-only publications because our review team lacked the language skills and resources to systematically and reliably evaluate non-English studies. This ensured consistency in our screening process and quality appraisal.

### Eligibility Criteria

We included randomized, non-randomized, and quasi-experimental studies – comparative (SDL vs. non-SDL or alternate SDL modalities) or pre-post designs – that evaluated author-defined SDL interventions (e.g., curricular or educational activities isolating self-directed methodologies) among health professions learners (medical students, residents, fellows; nursing students and nurse practitioners; dental students and residents; and allied health trainees such as physical therapy, pharmacy, and occupational therapy students). Studies included learners at all levels of health professions education, from undergraduate through postgraduate training. Eligible studies reported quantitative Kirkpatrick Level 2–4 outcomes (knowledge, skills, behaviors) [14] in English full-text manuscripts; we excluded veterinary or non-health undergraduates, licensed professionals learning outside formal programs (e.g., CME courses, procedural orientations), industry-led trainings, mixed SDL/non-SDL interventions without isolatable SDL, Level 1 only outcomes such as those that measure readiness for SDL, reviews, commentaries, case studies, qualitative-only studies, and abstract-only reports. We accepted synonymous terminology (independent learning, self-instruction, autonomous learning) but required clear isolation of the SDL component.

### Study Selection

**Figure 1:**
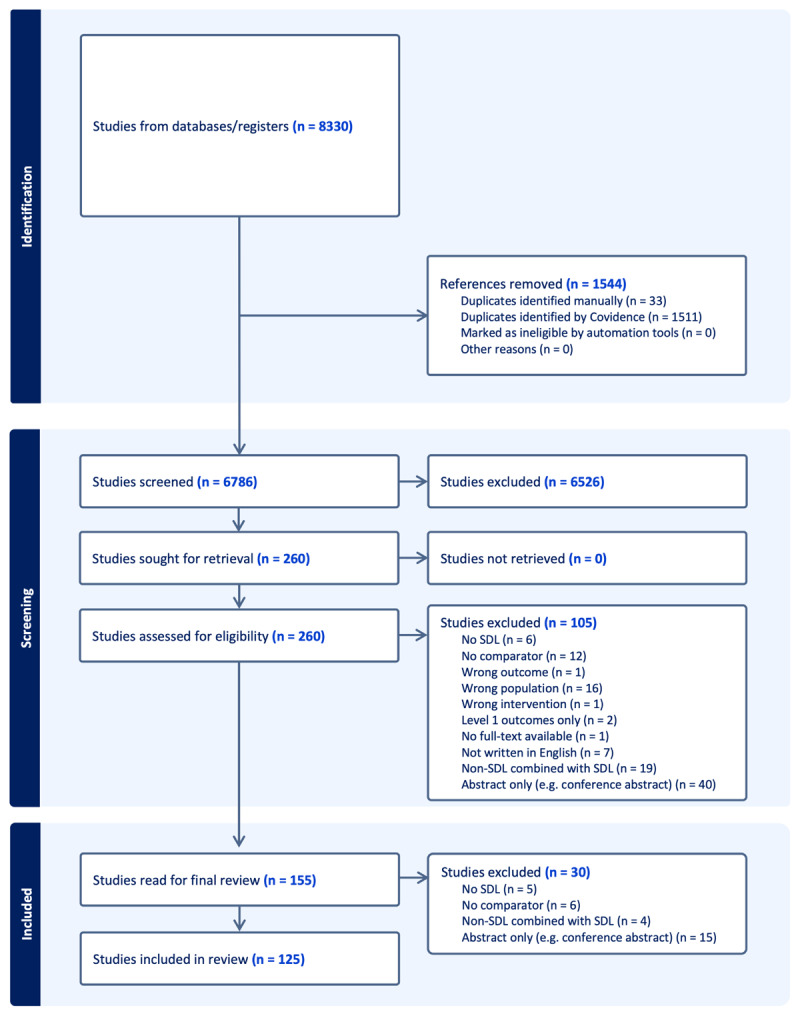
PRISMA flow diagram showing the study selection process for the systematic review and meta-analysis of self-directed learning in health professions education. The diagram illustrates the identification, screening, eligibility assessment, and inclusion phases, documenting reasons for exclusion at each stage.

We conducted a systematic review and meta-analysis that involved four steps: screening of titles and abstracts, full-text review, extraction, and analysis (Figure 1). We utilized COVIDENCE, a knowledge synthesis software, for screening, full-text review, and data extraction. Screening was conducted by a team of five researchers, with each article screened by at least two researchers independently. Conflicts were resolved by a third reviewer or through group consensus. We then performed a full-text review to determine final eligibility. Conflicts were resolved by a third independent reviewer or group consensus. Extraction followed in two parts, one for the systematic review and a second extraction of data specifically for the purposes of meta-analysis.

### Data Extraction

We collaboratively designed a data extraction template based on Murad et al.’s previous review and informed by our experiences as health professions educators and our familiarity with the recent literature from the earlier stages of the review. Variables extracted for the systematic review included: learner type, study design, learning modalities used for SDL and for the comparator, whether the teacher was absent or played a role in SDL, whether the self-directed learner was involved in choosing resources and in assessment, outcome type based on the Kirkpatrick model, [24] the aim of the study, funding and conflicts of interest. For the meta-analysis, additional variables extracted included sample size, study design, whether SDL was the experimental variable or a control, outcome measure, effect size (Cohen’s d), confidence interval, variance, and standard error. As a proof of concept for our research team, in an effort to facilitate future research efforts and evaluate the reliability of software, some portions of the extraction were also conducted in parallel using AI research software Elicit (https://elicit.com). However, this served as a supplementary exercise and all content was ultimately extracted manually by independent reviewers. Five independent reviewers participated in screening and extraction. Each article was screened by two independent reviewers. Likewise, two independent reviewers extracted data from each article. Discrepancies were resolved by consensus. For the meta-analysis, two reviewers conducted the data extraction and data analysis, and a third independent reviewer checked their work.

### Quality Appraisal

We systematically assessed the methodological quality of all included studies using the Medical Education Research Study Quality Instrument (MERSQI), a validated 10-item tool specifically designed to evaluate the quality of medical education research [25,26]. The MERSQI assesses six domains: (1) study design (single-group cross-sectional or single-group post-test only = 1 point; single-group pre-post or nonrandomized two-group = 2 points; randomized controlled trial = 3 points); (2) sampling, which evaluates both the number of institutions (single institution = 0.5 points; two institutions = 1 point; >2 institutions = 1.5 points) and response rate (not applicable = 0.5 points; <50% or not reported = 0 points; 50–74% = 0.75 points; ≥75% = 1.5 points); (3) type of data (assessment by study participant = 1 point; objective measurement = 3 points); (4) validity of evaluation instrument (not applicable = 3 points; instrument reported but validity evidence not reported = 1 point; validity evidence reported = 3 points); (5) data analysis (appropriate for study design and type of data = 3 points; beyond descriptive analysis = 2 points; descriptive analysis only = 1 point); and (6) outcomes assessed (Kirkpatrick Level 1 [satisfaction, attitudes, perceptions] = 1 point; Level 2 [knowledge, skills] = 1.5 points; Level 3 [behaviors] = 2 points; Level 4 [patient/system outcomes] = 3 points). The MERSQI yields a total score ranging from 5 to 18, with higher scores indicating higher methodological quality.

For each study, two of all five independent reviewers (SW, LM, PM, JM, BZ), conducted all quality appraisals. Each reviewer independently scored each study across all MERSQI domains, and disagreements were resolved through discussion and, when necessary, consultation with a third reviewer. MERSQI scores were documented in a structured data collection sheet. We did not exclude studies based on quality scores; instead, we used these assessments to characterize the overall quality of the evidence base.

Although we did not use a dedicated risk-of-bias instrument, several MERSQI domains correspond to bias domains in education and clinical research. For example, the study design domain relates to selection and allocation bias (randomization, comparison groups), the sampling and response-rate domains reflect risks of selection and nonresponse bias, the data-type and instrument-validity domains address detection and measurement bias, and the analysis domain corresponds to risks of selective reporting or inappropriate analytic decisions. Thus, while MERSQI does not provide a full risk-of-bias judgment as defined in PRISMA 2020, it captures many of the methodological features that function as proxies for risk-of-bias in medical education research.

### Meta-analysis process

To select eligible studies for meta-analysis, the study followed more restrictive inclusion criteria: 1. Studies had an experimental group and a control group; 2. Studies had sufficient data to compute effect sizes; 3. SDL was treated as either experimental or comparator. As a result, an additional 77 studies were excluded from the analysis. Two authors (BZ and JM) conducted the selection process of the meta-analysis and effect size computation.

Our meta-analysis used Cohen’ d as the effect size measure. To compute Cohen’s d, the components for calculating standardized mean differences were extracted [27]. Cohen’s d was calculated as the difference in posttest scores between the experimental and control groups divided by the pooled standard deviation at posttest [28]. In cases where studies did not report mean and standard deviation directly, we used appropriate conversion formulas (e.g., from t-values, F-ratios, or other statistical information) to estimate Cohen’s d when feasible [29].

After effect size standardization and transformation, a three-level meta analysis (3LM) was conducted using the rma.mv function in the R package metafor [30]. This modeling approach was chosen to address the hierarchical structure of the data, where individual effect sizes are nested within studies. By incorporating random effects at both the study level and the effect size level, the model accounts for the dependency among effect sizes that arises when multiple outcomes are reported within a single study. Meta-regressions were further performed to explore potential moderating effects of study-level or effect size-level variables.

### Ethics

Ethical approval was not required for this systematic review as it involved analysis of published literature only, with no primary data collection from human participants.

## Results

We identified 6,786 studies, of which 125 studies were included in the final analysis (See Appendix A). Forty-nine articles met the criteria for meta-analysis. Studies included trainees in medicine (n = 94), nursing (n = 15) dentistry (n = 7), and in other health professions (n = 6). In 98 studies, learning outcomes were categorized as Kirkpatrick level 2 outcomes, those that involve the measurement of knowledge or skills (typically through tests or formal assessments), while in 27 studies they were categorized as level 3, those that involve behavioral outcomes such as clinical performance. We did not identify any studies at Level 4 (Table 1). Study types included 91 randomized controlled trials, 17 non-randomized experimental studies, and 14 quasi-experimental studies. The most common SDL learning modalities utilized in these studies included written material (45 studies) followed by virtual reality and simulators (39 studies), video-based interventions (34 studies), and online or computer modules (32 studies). In most studies, SDL occurred in the absence of a teacher (77 studies), while a teacher served as a facilitator in 39 studies or as a content source in 9 studies. In most studies, learners were not involved in either choosing resources (99 studies) or in assessment (94 studies).

**Table 1:** Characteristics of studies included in a systematic review and meta-analysis of self-directed learning in health professions education (n = 125).


CHARACTERISTIC		N (%)

**Kirkpatrick**	Level 2	98 (78%)

	Level 3	27 (22%)

	Level 4	0 (0%)

**Study Type**	Randomized Controlled Trial	91 (73%)

	Non-Randomized Experimental	17 (14%)

	Quasi-Experimental	14 (11%)

	Other	3 (2%)

**Country**	Other	38 (30%)

	United States	34 (27%)

	Germany	10 (8%)

	India	10 (8%)

	Canada	8 (6%)

	Australia	6 (5%)

	China	6 (5%)

	Denmark	4 (3%)

	South Korea	4 (3%)

	United Kingdom	3 (2%)

**Learner Type**	Medicine	94 (75%)

	Nursing	15 (12%)

	Dentistry	7 (6%)

	Other	6 (5%)

	Pharmacy	3 (2%)

**SDL Modalities**	Textbook or Other Written Material	45 (36%)

	Other	43 (34%)

	Virtual Reality, Computer Simulation, or Physical Simulator	39 (31%)

	Video	34 (27%)

	Online Module or Computer Module	32 (26%)

	Real Patient Encounter	4 (3%)

**Teacher Role**	Absent	77 (62%)

	Facilitator	39 (31%)

	Content Source	9 (7%)

**Learner Involved in Choosing Resources?**	No	99 (79%)

	Yes	22 (18%)

**Learner Involved in Assessment?**	No	94 (75%)

	Yes	31 (25%)


### Meta-Analysis Results

A three-level random-effects meta-analysis model was adopted to account for the hierarchical structure of the data, where level 1 represents the sampling variance of the effect sizes, and level 2 captures the variability among effect sizes nested within studies, and level 3 accounts for variability between studies. A total of 48 studies [20,21,31,32,33,34,35,36,37,38,39,40,41,42,43,44,45,46,47,48,49,50,51,52,53,54,55,56,57,58,59,60,61,62,63,64,65,66,67,68,69,70,71,72,73,74,75,76], comprising 74 effect sizes, were included in the meta-analysis. When accounting for both within-study and between-study variability, the estimated average effect size was 0.34 (SE = 0.15, 95% CI [0.04, 0.64]), which was statistically significant (*p* = .0268), indicating a small to moderate overall effect of SDL (see supplementary material for the full Forest Plot).

**Figure 2:**
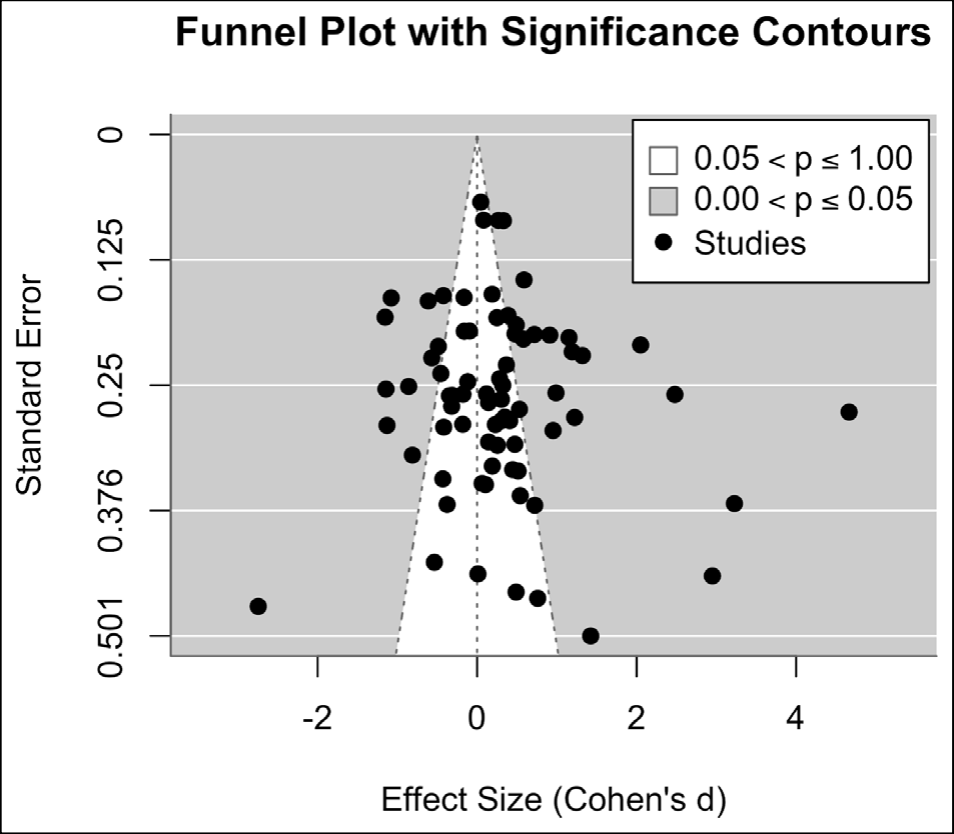
A funnel plot of the 74 effect sizes included in the meta-analysis on self-directed learning in health professions education.

Figure 2 displays a funnel plot of the 74 effect sizes included in the meta-analysis. The plot exhibits a generally symmetrical distribution around the pooled effect size, suggesting no clear evidence of publication bias or small-study effects. While a few studies appear outside the pseudo–95% confidence limits, the overall shape of the funnel remains well-balanced. This visual inspection does not raise substantial concerns regarding selective reporting. We conducted Egger’s regression test using a two-level random-effects model. The test result was non-significant (t(72) = 1.05, p = .297), indicating no statistical evidence of small-study effects or publication bias. The limit estimate as the standard error approaches zero was 0.03 (95% CI [–0.36, 0.41]), further supporting the conclusion that asymmetry is not a major concern in this meta-analysis.

### Moderator Analysis

**Figure 3:**
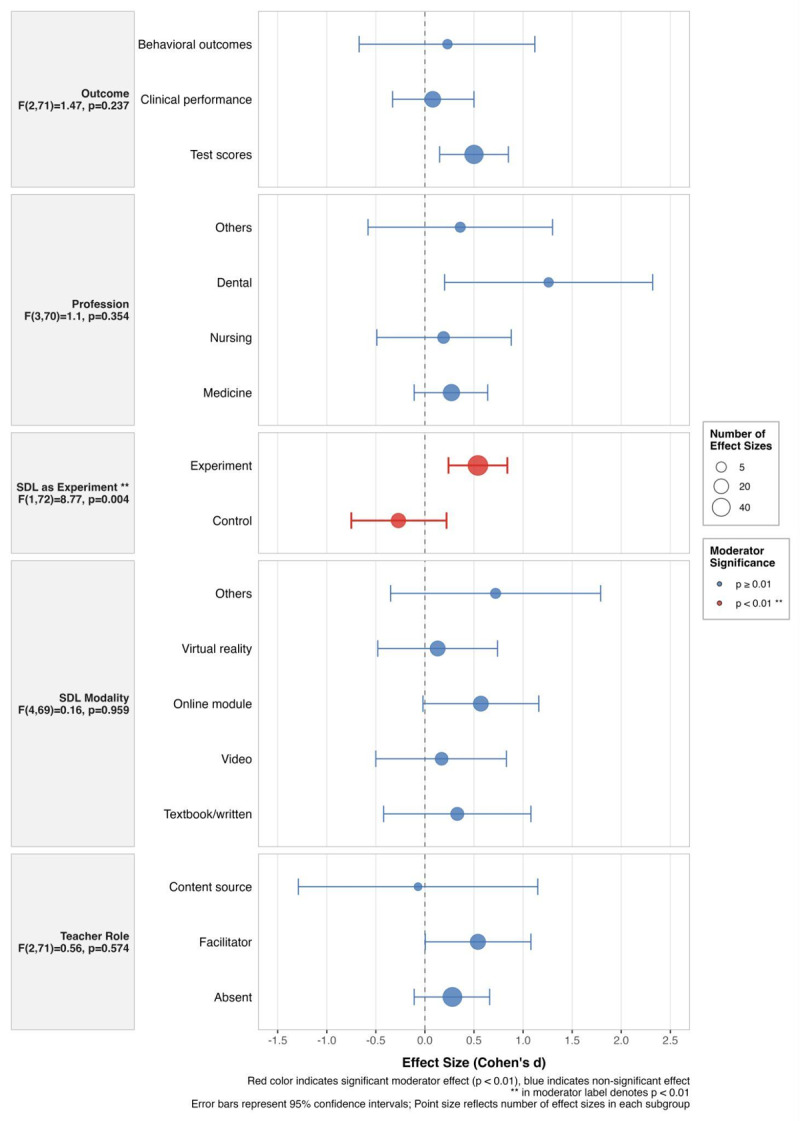
Forest plot of effect sizes by moderators.

Meta-regressions were performed to investigate the potential moderating effects of profession, SDL as experimental, outcome type, SDL modality, and teacher role on effect size estimates (see Table 2 and Figure 3).

**Table 2:** Moderator analysis results.


SUBGROUP/DOMAIN	EFFECT SIZES (n)	STUDIES (k)	COHEN'S *d*	95% CI	r	*F*	*df*	*p*

**Professions**						1.1	3, 70	0.354

Medicine	30	47	0.27	[–0.11, 0.64]	0.26			

Nursing	9	17	0.19	[–0.49, 0.88]	0.19			

Dental	4	4	1.26	[0.20, 2.32]	0.85			

Others	5	6	0.36	[–0.58, 1.30]	0.35			

**SDL as experiment**						8.77	1, 72	0.004

Control	19	13	–0.27	[–0.75, 0.22]	–0.26			

Experiment	55	36	0.54	[0.24, 0.84]	0.50			

**Outcome**						1.47	2, 71	0.237

Test scores	44	30	0.50	[0.15, 0.85]	0.46			

Clinical performance	26	21	0.08	[–0.33, 0.50]	0.08			

Behavioral outcomes	4	3	0.23	[–0.67, 1.12]	0.22			

**SDL modality**						0.16	4, 69	0.759

Textbook or other written material	13	8	0.33	[–0.42, 1.08]	0.32			

Video	12	11	0.17	[–0.50, 0.83]	0.16			

Online module or computer module	22	13	0.57	[–0.019, 1.161]	0.52			

Virtual reality	22	12	0.13	[–0.48, 0.74]	0.13			

Others	5	4	0.72	[–0.35, 1.79]	0.62			

**Teacher role**						0.56	2, 71	0.574

Absent	48	30	0.28	[–0.11, 0.66]	0.27			

Facilitator	23	15	0.54	[0.004, 1.083]	0.50			

Content source	3	3	–0.07	[–1.29, 1.15]	–0.07			


In terms of professional contexts, although not statistically significant (F(3, 70) = 1.10, p = 0.354), the largest effects were observed in dental education (d = 1.26, 95% CI [0.20, 2.32], r = 0.85), followed by other professions (d = 0.36, 95% CI [–0.58, 1.30], r = 0.35), medicine (d = 0.27, 95% CI [–0.11, 0.64], r = 0.26), and nursing (d = 0.19, 95% CI [–0.49, 0.88], r = 0.19).

SDL treated as experimental groups showed a significantly larger effect (d = 0.54, 95% CI [0.24, 0.84], r = 0.50) compared to comparator groups (d = –0.27, 95% CI [–0.75, 0.22], r = –0.26), with this difference being statistically significant (F(1, 72) = 8.77, p = 0.004).

The type of outcome measure did not significantly moderate the effect sizes (F(2, 71) = 1.47, p = 0.237). Test scores showed the largest effect (d = 0.50, 95% CI [0.151, 0.854], r = 0.46), followed by behavioral outcomes (d = 0.23, 95% CI [–0.67, 1.12], r = 0.223) and clinical performance (d = 0.08, 95% CI [–0.33, 0.50], r = 0.08).

SDL modality analysis revealed no significant differences between delivery methods (F(4, 69) = 0.16, p = 0.759). The largest effects were observed for others (d = 0.72, 95% CI [–0.35, 1.79], r = 0.62), followed by online modality (d = 0.57, 95% CI [–0.02, 1.16], r = 0.52), textbook or other print (d = 0.33, 95% CI [–0.42, 1.08], r = 0.32), video (d = 0.17, 95% CI [–0.50, 0.83], r = 0.16), and virtual reality (d = 0.13, 95% CI [–0.48, 0.74], r = 0.13).

For teacher role, no significant moderation effect was detected (F(2, 71) = 0.56, p = 0.574). The facilitator role showed the largest effect (d = 0.54, 95% CI [0.004, 1.083], r = 0.50), followed by absent (d = 0.28, 95% CI [–0.11, 0.66], r = 0.27), while content source showed a slight negative effect (d = –0.07, 95% CI [–1.29, 1.15], r = –0.07).

### Quality Appraisal Results

The 125 included studies demonstrated moderate overall methodological quality based on MERSQI assessment. Mean MERSQI score was 12.65 (SD = 1.23, range 10–15.5). Twenty- six studies (21%) were rated as high quality (≥14 points), 99 studies (79%) as average quality (10–13.5 points), and 0 studies (0%) as low quality (<10 points).

Across individual MERSQI domains, specific findings were as follows: (1) Study design: 73% of studies used RCTs (3 points), 15% used nonrandomized comparative designs (2 points), and 12% used single-group pre-post test designs (1.5 points), none (0%) used single group cross-sectional or post-test designs (1 point). (2) Sampling: 2% were conducted at >2 institutions (1.5 points), 5% at 2 institutions (1 point), and 93% at single institutions (0.5 points). Response rates were ≥75% in 72% of studies, 50–74% in 7%, and <50% or unreported in 21%. (3) Type of data: 100% used objective measurements (3 points) while 0% relied on participant self-assessment (1 point). (4) Validity evidence: 2% reported three measures of validity for outcome instruments (3 points), 14% reported two measures of validity for outcome instruments (2 points), 26% reported one measure of validity for outcome instruments (1 point), and 58% reported no validity measures. (5) Data analysis: 100% performed analyses beyond descriptive statistics (2 points), while 0% used descriptive analysis only (1 point). (6) Outcomes: 0% assessed Kirkpatrick Level 1 outcomes only, 78% level 2 outcomes (knowledge/skills; 1.5 points), 22% Level 3 outcomes (behaviors; 2 points), and 0% Level 4 outcomes (patient/system; 3 points).

The MERSQI domains highlight several potential sources of bias across included studies. The predominance of randomized controlled trials and the universal use of objective outcome measures reduce concerns about performance and detection bias. However, the high proportion of single-institution samples and limited reporting of validity evidence for outcome instruments suggest possible risks of selection bias and measurement bias.

## Discussion

In this systematic review and meta-analysis, we updated and extended the work of Murad et al. [1]. by examining the effects of SDL on knowledge, clinical performance, and behavioral outcomes in HPE.

The final selection of 125 studies draw heavily from medical education, with comparatively fewer investigations in nursing, dentistry, and other health professions. Most of these studies focused on Kirkpatrick level 2 outcomes, suggesting that knowledge and skills remain the most commonly assessed impacts of SDL; indeed, the complete absence of Kirkpatrick level 4 studies may reflect the challenges in measuring patient or system-level outcomes in health professions education. It is also notable that while teacher absence was frequently reported (aligning with the general expectations of SDL described by Knowles) learners were rarely involved in assessments or choosing resources, which raises questions about how “self-directed” many interventions truly are.

We acknowledge that our analysis of three SDL components (educator role, learner resource selection, and learner self-assessment) represents a pragmatic compromise rather than a comprehensive assessment of Knowles’ full model. Following Murad et al.’s approach, we selected these three components because they were the most consistently and explicitly reported elements in SDL studies. As Murad et al. previously noted, these components were present in 55%, 50%, and 70% of published SDL studies respectively, and thus could be most reliably coded across studies. While Knowles’ complete framework encompasses five interconnected processes: (1) diagnosing learning needs, (2) formulating learning goals, (3) identifying resources for learning, (4) choosing and implementing learning strategies, and (5) evaluating learning outcomes, the vast majority of studies do not report sufficient detail about learners’ involvement in needs diagnosis or goal formulation to permit systematic coding. This gap between SDL’s theoretical foundation and its operational reporting in research studies represents a critical challenge for the field and underscores the need for more comprehensive and transparent reporting in future SDL intervention studies.

Our meta-analyses, which included 48 studies and 74 effect sizes, revealed a small-to-moderate overall effect of SDL when compared with more traditional or alternative instructional strategies. Considerable heterogeneity was observed across studies, reflecting the varied professional disciplines, diverse SDL modalities, and differing degrees of facilitator involvement.

The combination of small-to-moderate effects and high heterogeneity suggests that SDL’s effectiveness is not uniform—its success may depend on a variety of factors such as the quality of SDL implementation or how well the SDL approach is tailored to specific educational contexts. These findings underscore that SDL should not be treated as a one-size-fits-all solution; instead, educators must carefully align instructional design with learner needs, content complexity, and institutional support systems to maximize impact.

A central finding of this review is the disconnection between SDL as theorized and SDL as implemented. Although many interventions were labeled as SDL, only 21% of studies involved learners in resource selection and 25% in assessment, both of which are core components of Knowles’ definition. This implementation gap suggests that many interventions labeled as “SDL” may not fully embody the principles of self-direction. Such incomplete reporting and partial implementation may help explain both the modest effect sizes observed and the apparent lack of progress in SDL research since Murad et al. (2010).

Our moderator analyses did not identify definitive explanatory factors beyond group assignment (i.e. whether or not SDL was the experimental condition). Studies in which SDL was used as the experimental condition yielded significantly higher effect sizes, which suggests that SDL may be particularly effective when it is introduced with intention as an active intervention compared to traditional or instructor-led methods.

The residual variability suggests that the success of SDL may depend on other additional implementation factors that were not consistently reported across studies. These include the quality and timeliness of feedback, the presence of structured learner engagement strategies, the alignment of SDL activities with learners’ developmental readiness, and the broader educational context, including institutional support and psychological safety [77,78].

Without adequate reporting on these contextual and pedagogical elements, it remains difficult to disentangle which components of SDL contribute most to its effectiveness.

Our findings are broadly consistent with the earlier review by Murad et al., which likewise reported moderate gains in measured outcomes [1]. However, our study extends their work in several important ways: we include a larger and more recent sample of studies across a broader range of health professions, apply a three-level meta-analytic model to better account for hierarchical data structure, and examine key moderating factors such as professional discipline, teacher role, and SDL modalities. These additions allow for a more granular understanding of how SDL functions across contexts and highlight the importance of intentional, well-designed SDL interventions. The updated evidence suggests that SDL interventions continue to be an effective strategy in promoting learner autonomy and knowledge attainment. Moreover, our results align with adult learning theories (e.g., Knowles’s andragogy model) and the Master Adaptive Learner framework by highlighting the value of learner-driven education [2]. Yet, our analysis points to potential limitations of “teacher-absent” SDL, particularly for developing clinical or behavioral competencies, reinforcing the idea that structured support, facilitation, and timely feedback may be critical to translate knowledge gains into real-world practice.

Beyond highlighting the effectiveness of SDL as a general instructional strategy, our findings point to the importance of modality-specific considerations. The way in which SDL is implemented, whether through asynchronous online modules, blended learning environments, virtual simulations, or face-to-face formats, can substantially shape learner engagement and outcomes. For instance, online SDL modules may promote flexibility and access to diverse resources, but can also risk disengagement without timely feedback [35]. In contrast, simulation-based SDL, particularly when supported by deliberate practice and debriefing, may be better suited for developing non-technical competencies, such as communication skills [30]. Therefore, future SDL interventions must carefully consider how modality interacts with learning goals, facilitator involvement, and feedback mechanisms to optimize educational impact.

Based on our synthesis, several practical recommendations emerge for implementing SDL in HPE research. First, interventions should be labeled as SDL only when learners have genuine autonomy in both resource selection and self-assessment (core components that were absent in the majority of the studies we reviewed). Second, SDL interventions are likely to yield only modest knowledge gains unless they are carefully designed and implemented deliberately as the primary educational strategy, as our analysis showed significantly stronger effects when SDL was the intentional experimental intervention rather than a control condition. Third, SDL appears most appropriate for knowledge acquisition rather than skill development, given the stronger effects observed for test scores compared to clinical performance outcomes. Finally, while many interventions eliminated teacher involvement entirely (62% of studies), our findings suggest that structured facilitator support could enhance SDL effectiveness, though this trend did not reach statistical significance. Future studies should test facilitation models that balance learner autonomy with targeted guidance to optimize SDL’s impact.

A key strength of this review is the inclusion of both randomized and non-randomized studies spanning multiple health professions and significant international representation, thus providing a broad and updated perspective on SDL interventions.

We used the MERSQI for our systematic quality appraisal because it is a widely validated and field-specific appraisal instrument in HPE. Although MERSQI does not generate categorical risk-of-bias judgments, several of its domains align with conventional bias constructs (e.g., selection bias via study design and sampling; detection bias via data type and validity evidence; reporting bias via analytic rigor). Therefore, our quality appraisal provides an indirect but meaningful assessment of bias in the included studies.

The MERSQI findings informed our interpretation of the results. They indicated that included studies demonstrated moderate overall methodological quality, which is consistent with the broader medical education literature, and helped to ensure that only moderate-to-high quality studies were included in the review, with a predominance of randomized controlled trials.

The characterization of quality limitations, particularly the predominance of single-institution designs, high variability in the reporting of validity measures, and dominant focus on knowledge-level outcomes (i.e. Kirkpatrick Level 2), helps to contextualize the substantial heterogeneity observed and tempers the confidence with which strong causal conclusions can be drawn.

Several other key limitations also warrant consideration.

First, we focused on studies explicitly framed as SDL which, while ensuring conceptual clarity, excluded potentially relevant work from related frameworks such as self-regulated learning.

Second, there was marked heterogeneity in how “self-directed learning” was defined and implemented. Studies varied widely in learner populations, outcome measures (predominantly Kirkpatrick level 2), instructional modalities, and the role of facilitators or feedback mechanisms. This inconsistency makes cross-study comparisons challenging and raises questions about whether all interventions truly embodied core SDL principles (e.g., learner choice of resources, self-assessment).

Third, although Egger’s test did not detect significant publication bias, the possibility of underreported null or negative findings remains.

Fourth, our English-only search may have missed important non-English contributions.

Finally, reliance on published study data meant that many critical design features, such as the degree of facilitator involvement, structure and quality of feedback, the presence of scaffolded instructional pathways or mastery-based progression, and measures of intervention fidelity, were incompletely reported, limiting our ability to tease apart which SDL components drive observed effects.

### Future Directions

To advance understanding of SDL’s impact in HPE, future work should begin with standardized, theory-informed operational definitions of SDL and discipline-specific outcome measures with validity evidence, encompassing not only knowledge acquisition, but also skill performance, behavior change, and where feasible, patient-level outcomes. Authors must report intervention fidelity in detail, clarifying how learners select resources, how facilitators guide learning, and how feedback is delivered. Rigorous experimental designs that purposefully position SDL as the primary intervention (rather than a superficial comparator) will help isolate its true effect (notably, studies treating SDL as the experimental condition yielded substantially larger effect sizes). Even when SDL is used as a control, its implementation should be intentional, systematic, and well-defined. Research should seek to describe how curriculum designers align educational modalities with specific learning objectives (e.g., online modules for foundational knowledge, simulations or VR for procedural skills), and how they apply instructional-design principles. Finally, to capture the complexity and longitudinal impact of SDL, future research should employ diverse methodologies. Longitudinal designs can assess sustained changes in learner behavior. Mixed methods studies can offer deeper insights into learners’ experience, motivation, and engagement with SDL. Together, these approaches will illuminate which SDL components most effectively foster autonomy, adaptability, and ultimately, improved learning outcomes, and under what conditions.

## Conclusion

This updated meta-analysis reaffirms that SDL enhances knowledge acquisition but suggests that it may yield only modest gains in clinical skills and behaviors. More importantly, our findings highlight a persistent gap between SDL as theorized and SDL as implemented. Despite the central role of learner autonomy in Knowles’ model, most interventions lacked authentic opportunities for learners to select resources or engage in self-assessment, raising questions about whether they truly constituted SDL. The wide variability in how SDL is defined and reported underscores the need for a consensus definition of SDL, standardized operational frameworks and transparent and detailed intervention descriptions.

Practically, SDL is most likely to succeed when introduced intentionally as the primary instructional strategy, when facilitator support is balanced with learner autonomy, and when the modality aligns with the targeted learning outcomes. By closing the gap between SDL’s conceptual foundation and its applied implementation, educators and researchers can more fully realize its potential to foster autonomy, adaptability, and lifelong learning in HPE.

## Additional Files

The additional files for this article can be found as follows:

10.5334/pme.2128.s1 Supplementary material. Forest plot of effect sizes included in the meta-analysis on self-directed learning in health professions education.

10.5334/pme.2128.s2 Appendix A. List of Extracted Papers and Search Strategy.

## Disclaimer

The opinions and assertions expressed herein are those of the authors and do not necessarily reflect the official policy or position of the Uniformed Services University, the Department of Defense, or the Federal Government of the United States.
