# Supplementary material for: Self-Directed Learning in Health Professions Education: A Systematic Review and Meta-Analysis
Source: Perspect Med Educ. 2026 Jan 28;15(1):37–52. doi: 10.5334/pme.2128 (PMC12857615; doi:10.5334/pme.2128)

Appendix B

Supplementary material: Forest plot of effect sizes included in the meta-analysis on self-directed learning in health professions education

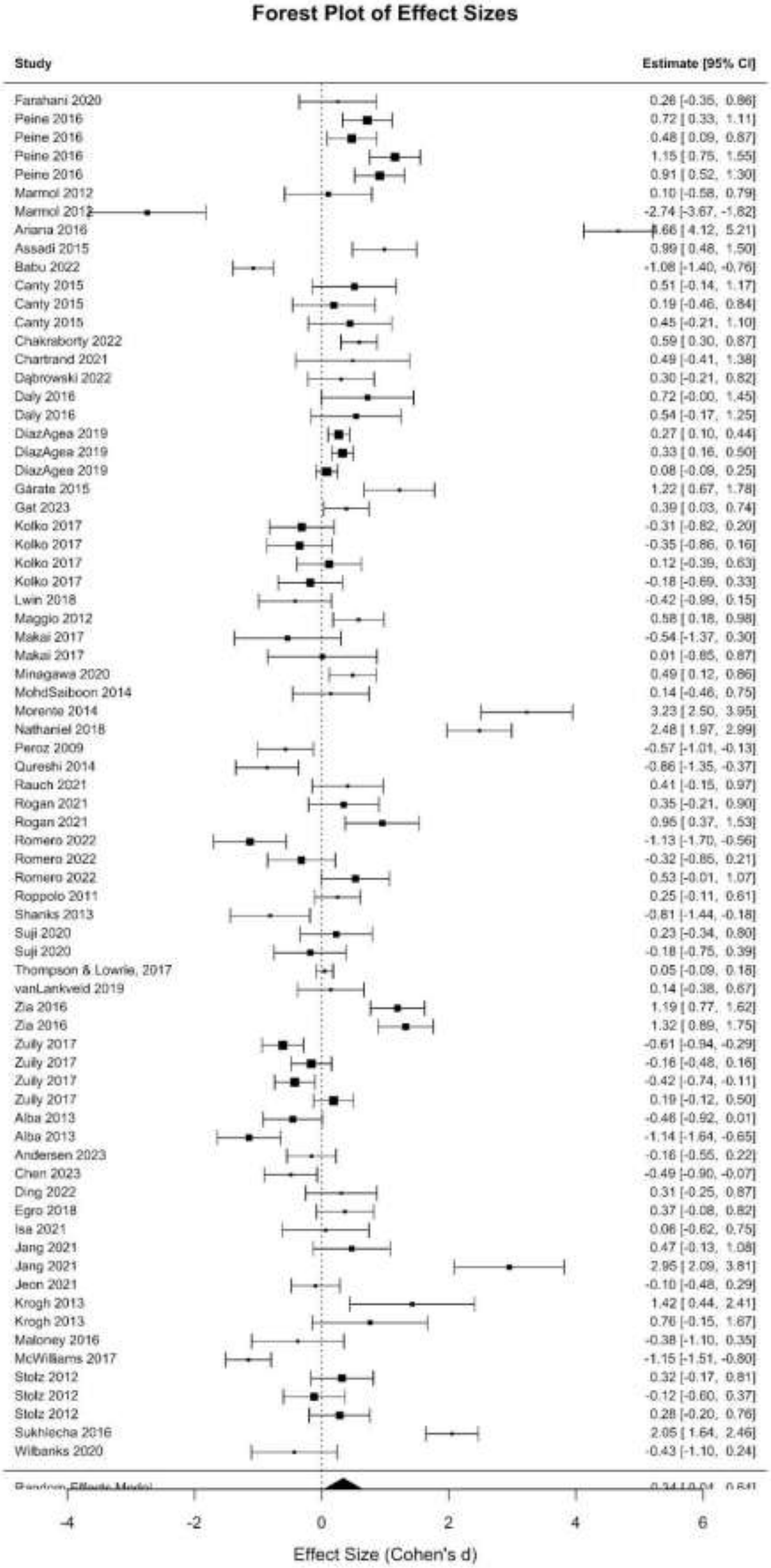

Supplement: Supplementary material. — Forest plot of effect sizes included in the meta-analysis on self-directed learning in health professions education. [file pme-15-1-2128-s1.pdf]
