# Supplementary material for: Self-Directed Learning in Health Professions Education: A Systematic Review and Meta-Analysis
Source: Perspect Med Educ. 2026 Jan 28;15(1):37–52. doi: 10.5334/pme.2128 (PMC12857615; doi:10.5334/pme.2128)
Supplement: Appendix A. — List of Extracted Papers and Search Strategy. [file pme-15-1-2128-s2.pdf]

Appendix A
List of Extracted Papers and Search Strategy

Papers included in meta-analysis

1. Alba GA, Kelmenson DA, Noble VE, Murray AF, Currier PF. Faculty staff-guided versus self-guided ultrasound training for internal medicine residents. Medical Education. 2013;47(11):1099-108.
2. Andersen NL, Jensen RO, Konge L, et al. Immersive Virtual Reality in Basic Point-of-Care Ultrasound Training: A Randomized Controlled Trial. Ultrasound Med Biol. 2023;49(1):178-185. doi:10.1016/j.ultrasmedbio.2022.08.012
3. Ariana A, Amin M, Pakneshan S, Dolan-Evans E, Lam AK. Integration of Traditional and E-Learning Methods to Improve Learning Outcomes for Dental Students in Histopathology. Journal of Dental Education. 2016;80(9):1140-8.
4. Assadi T, Mofidi M, Rezai M, Hafezimoghadam P, Maghsoudi M, Mosaddegh R, et al. The comparison between two methods of basic life support instruction: Video self-instruction versus traditional method. Hong Kong Journal of Emergency Medicine. 2015;22(5):291-6.
5. Babu R, Bahuleyan B, Panchu P, Shilpa AV, Sreeja CK, Manjuran DJ. Effectiveness of Self-paced and Instructor-led Online Learning: A Study among Phase I Medical Students. Journal of Clinical and Diagnostic Research. 2022;16(1):JC01-JC4.
6. Canty DJ, Hayes JA, Story DA, Royse CF. Ultrasound simulator-assisted teaching of cardiac anatomy to preclinical anatomy students: A pilot randomized trial of a three-hour learning exposure. Anatomical Sciences Education. 2015;8(1):21-30.
7. Chakraborty S, Chaudhuri A, Mukherjee A, Adhya D. A study to evaluate the outcomes of traditional lecture classes and self-directed learning sessions among the first MBBS students in the department of physiology. National Journal of Physiology, Pharmacy and Pharmacology. 2022;12(11):1880-6.
8. Chartrand G, Soucisse M, Dubé P, Trépanier JS, Drolet P, Sideris L. Self-directed learning by video as a means to improve technical skills in surgery residents: a randomized controlled trial. BMC medical education. 2021;21(1):91.
9. Chen L, Tang XJ, Liu Q, Zhang X. Self-directed learning: Alternative for traditional classroom learning in undergraduate ophthalmic education during the COVID-19 pandemic in China. HELIYON. 2023;9(5).
10. Crihfield EG, Uppalapati P, Abittan B, Laibangyang A, Brahmbhatt S, Burlingame M, et al. Development of laparoscopic skills in skills-naïve trainees using self-directed learning with take-home laparoscopic trainer boxes. Surgery Open Science. 2023;16:82-93.
11. Dąbrowski F, Stogowski P, Białek J, Fliciński F, Piotrowski M, Pankowski R, et al. Video-Based Microsurgical Education versus Stationary Basic Microsurgical Course: A Noninferiority Randomized Controlled Study. Journal of Reconstructive Microsurgery. 2022;38(7):585-92.
12. Daly C, Vennalaganti P, Soudagar S, Hornung B, Sharma P, Gupta N. Randomized controlled trial of self-directed versus in-classroom teaching of narrow-band imaging for

diagnosis of Barrett's esophagus-associated neoplasia. Gastrointestinal Endoscopy. 2016;83(1):101-6.
13. Díaz Agea JL, Megías Nicolás A, García Méndez JA, Adánez Martínez MG, Leal Costa C. Improving simulation performance through Self-Learning Methodology in Simulated Environments (MAES©). Nurse Education Today. 2019;76:62-7.
14. Ding JB, Yap ASJ, Thng ZX, Gan NY, Tan JCH, Yip CC. Investigating mental rehearsal's applicability in guiding independent E-learning (IMAGINE) of eye examination skills during the pandemic. Medical Teacher. 2022.
15. Egro FM, Tayler-Grint LC, Vangala SK, Nwaiwu CA. Multicenter Randomized Controlled Trial to Assess an e-Learning on Acute Burns Management. Journal of burn care & research : official publication of the American Burn Association. 2018;39(1):94-9.
16. Farahani S, Farahani I, Burckhardt BB, Schwender H, Laeer S. Self-instruction video versus face-to-face instruction of pharmacy students' skills in blood pressure measurement. Pharmacy. 2020;8(4):1-14.
17. Gárate IB, Berrotarán GB. Self-directed pharmacotherapy learning to fifth-year pharmacy students in Spain. Indian Journal of Pharmaceutical Education and Research. 2015;49(1):10-7.
18. Gat T, Galante O, Sadeh R, Kobal SL, Fuchs L. Self-learning of cardiac ultrasound by medical students: can augmented online training improve and maintain manual POCUS skills over time?. J Ultrasound. 2024;27(1):73-80. doi:10.1007/s40477-023-00804-5
19. Isa MH, Lim K, Jaafar MJ, Saiboon IM. The Effectiveness of Self-Instructional Video vs. Classroom Teaching Method on Focused Assessment With Sonography in Trauma Among House Officers in University Hospital. FRONTIERS IN SURGERY. 2021;8.
20. Jang K, Kim SH, Oh JY, Mun JY. Effectiveness of self-re-learning using video recordings of advanced life support on nursing students' knowledge, self-efficacy, and skills performance. BMC Nursing. 2021;20(1).
21. Jeon J, Park S. Self-Directed Learning versus Problem-Based Learning in Korean Nurse Education: A Quasi-Experimental Study. Healthcare (Basel). 2021;9(12):1763. Published 2021 Dec 20. doi:10.3390/healthcare9121763
22. Kolko RP, Kass AE, Hayes JF, Levine MD, Garbutt JM, Proctor EK, et al. Provider Training to Screen and Initiate Evidence-Based Pediatric Obesity Treatment in Routine Practice Settings: A Randomized Pilot Trial. Journal of pediatric health care : official publication of National Association of Pediatric Nurse Associates & Practitioners. 2017;31(1):16-28.
23. Krogh CL, Konge L, Bjurström J, Ringsted C. Training on a new, portable, simple simulator transfers to performance of complex bronchoscopy procedures. CLINICAL RESPIRATORY JOURNAL. 2013;7(3):237-44.
24. Lwin AT, Lwin T, Naing P, Oo Y, Kidd D, Cerullo M, et al. Self-Directed Interactive Video-Based Instruction Versus Instructor-Led Teaching for Myanmar House Surgeons: A Randomized, Noninferiority Trial. Journal of Surgical Education. 2018;75(1):238-46.
25. Maggio MP, Hariton-Gross K, Gluch J. The use of independent, interactive media for education in dental morphology. Journal of Dental Education. 2012;76(11):1497-511.
26. Makai GE, Schaeffer KD, Sloan NL. Independent learning of electrosurgery in gynecology: A randomized controlled trial. Journal of Gynecologic Surgery. 2017;33(2):51-6.

27. Maloney E, Hippe DS, Paladin A, Chew FS, Ha AS. Musculoskeletal Ultrasound Training for Radiology Residents: Lecture Versus Interactive Learning Module. Academic Radiology. 2016;23(7):789-96.
28. Marmol MT, Braga F, Garbin LM, Moreli L, dos Santos CB, de Carvalho EC. Central catheter dressing in a simulator: the effects of tutor's assistance or self-learning tutorial. REVISTA LATINO-AMERICANA DE ENFERMAGEM. 2012;20(6):1134-41.
29. Minagawa A, Mikoshiba Y, Koga H, Okuyama R. Dermoscopy image-based self-learning on computer improves diagnostic performance of medical students compared with classroom-style lecture in ultra-short period. Journal of Dermatology. 2020;47(12):1432-5.
30. Mohd Saiboon I, Jaafar MJ, Ahmad NS, Nasarudin NM, Mohamad N, Ahmad MR, et al. Emergency skills learning on video (ESLOV): A single-blinded randomized control trial of teaching common emergency skills using self-instruction video (SIV) versus traditional face-to-face (FTF) methods. Medical Teacher. 2014;36(3):245-50.
31. Morente L, Morales-Asencio JM, Veredas FJ. Effectiveness of an e-learning tool for education on pressure ulcer evaluation. Journal of Clinical Nursing. 2014;23(13-14):2043-52.
32. Nathaniel TI, Gainey JC, Williams JA, Stewart BL, Hood MC, Brechtel LE, et al. Impact and educational outcomes of a small group self-directed teaching strategy in a clinical neuroscience curriculum. Anatomical Sciences Education. 2018;11(5):478-87.
33. Peine A, Kabino K, Spreckelsen C. Self-directed learning can outperform direct instruction in the course of a modern German medical curriculum - results of a mixed methods trial. BMC medical education. 2016;16:158.
34. Peroz I, Beuche A, Peroz N. Randomized controlled trial comparing lecture versus self studying by an online tool. Medical Teacher. 2009;31(6):508-12.
35. Qureshi A, Rizvi F, Syed A, Shahid A, Manzoor H. The method of loci as a mnemonic device to facilitate learning in endocrinology leads to improvement in student performance as measured by assessments. Advances in physiology education. 2014;38(2):140-4.
36. Rauch A, Jahn F, Roesner A, Hahnel S, Schierz O. Impact of the DC/TMD instructional video on the practical skills of undergraduate students-A single-blinded, randomized controlled trial. European journal of dental education : official journal of the Association for Dental Education in Europe. 2021;25(3):435-41.
37. Rogan S, Taeymans J, Zuber S, Zinzen E. Impact of guided self-study on learning success in undergraduate physiotherapy students in Switzerland - a feasibility study of a higher education intervention. BMC medical education. 2021;21(1):362.
38. Romero P, Gerhaeuser A, Carstensen L, Kössler-Ebs J, Wennberg E, Schmidt MW, et al. Learning of Intracorporal Knot Tying in Minimally Invasive Surgery by Video or Expert Instruction. European Journal of Pediatric Surgery. 2022;33(3):228-33.
39. Roppolo LP, Heymann R, Pepe P, Wagner J, Commons B, Miller R, et al. A randomized controlled trial comparing traditional training in cardiopulmonary resuscitation (CPR) to self-directed CPR learning in first year medical students: The two-person CPR study. Resuscitation. 2011;82(3):319-25.
40. Shanks D, Brydges R, den Brok W, Nair P, Hatala R. Are two heads better than one? Comparing dyad and self-regulated learning in simulation training. Medical Education. 2013;47(12):1215-22.

41. Stolz D, Langewitz W, Meyer A, Pierer K, Tschudi P, S'ng CT, et al. Enhanced Didactic Methods of Smoking Cessation Training for Medical Students-A Randomized Study. Nicotine and Tobacco Research. 2012;14(2):224-8.
42. Suji M, Ramesh S, Devi S, Prasad D, Rajan L. Recalling the definitions with a mind mapping and self-learning technique: A comparative study in undergraduate students. Indian Journal of Forensic Medicine and Toxicology. 2020;14(4):3668-73.
43. Sukhlecha A, Jadav SP, Gosai TR, Balusamy D. Student-led objective tutorials in Pharmacology: An interventional study. Indian Journal of Pharmacology. 2016;48(Suppl 1):S83-S8.
44. Thompson AR, Lowrie DJ. An evaluation of outcomes following the replacement of traditional histology laboratories with self-study modules. Anatomical Sciences Education. 2017;10(3):276-85.
45. Van Lankveld W, Maas M, van Wijchen J, Visser V, Staal JB. Self-regulated learning in physical therapy education: a non-randomized experimental study comparing self-directed and instruction-based learning. BMC medical education. 2019;19(1):50.
46. Wilbanks BA, McMullan S, Watts PI, White T, Moss J. Comparison of Video-Facilitated Reflective Practice and Faculty-Led Debriefings. Clinical Simulation in Nursing. 2020;42:1-7.
47. Zia S, Jabeen F, Atta K, Sial NA. Self Directed Learning (SDL), an effective method for teaching physiology to medical students. Pakistan Journal of Medical and Health Sciences. 2016;10(3):700-3.
48. Zuily S, Phialy L, Germain E, Unlu O, Dufrost V, Clerc-Urmès I, et al. Impact of antiphospholipid syndrome iBook on medical students' improvement of knowledge: An international randomized controlled experimental study. Arthritis and Rheumatology. 2017;69.

Papers not included in meta-analysis

1. Abugideiri M, Schreibmann E, Switchenko J, McDonald MW, Beitler JJ, Curran WJ, et al. A Prospective International Pilot Study Evaluating the Efficacy of a Self-Guided Contouring Teaching Module with Integrated Feedback for Transitioning from 2D to 3D Treatment Planning. International Journal of Radiation Oncology Biology Physics. 2019;103(5):E6.
2. Agrawal P, Verma N. Prediscussion and Postdiscussion Assessment Scores in a Self-directed Learning Module Implemented in the Department of Biochemistry: A Comparative Study. Indian Journal of Medical Specialities. 2020;11(2):81–4.
3. Allen JE, Vennalaganti P, Gupta N, Hornung B, Alsop BR, Lim D, et al. Randomized controlled trial of self-directed vs. in-classroom education of Narrow Band Imaging (NBI) in diagnosing colorectal polyps using the NICE criteria. Gastroenterology. 2015;148(4):S150–1.
4. Andersen SAW, Foghsgaard S, Konge L, Caye-Thomasen P, Sorensen MS. The effect of self-directed virtual reality simulation on dissection training performance in mastoidectomy. The Laryngoscope. 2016;126(8):1883–8.

5. Axt S, Storz P, Ehrenberg C, Falch C, Immenroth M, Kirschniak A, et al. Evaluation of self-educational training methods to learn laparoscopic skills - a randomized controlled trial. BMC medical education. 2018;18(1):85.
6. Bernard A, Chemaly P, Dion F, Laribi S, Remerand F, Angoulvant D, et al. Evaluation of the efficacy of a self-training programme in focus cardiac ultrasound with simulator. Archives of cardiovascular diseases. 2019;112(10):576–84.
7. Bobby Z, Meiyappan K. “Test-enhanced” focused self-directed learning after the teaching modules in biochemistry. BIOCHEMISTRY AND MOLECULAR BIOLOGY EDUCATION. 2018;46(5):472–7.
8. Boody BS, Hashmi SZ, Rosenthal BD, Maslak JP, McCarthy MH, Patel AA, et al. The Effectiveness of Bioskills Training for Simulated Lumbar Pedicle Screw Placement. Global Spine Journal. 2018;8(6):557–62.
9. Brydges R, Carnahan H, Rose D, Dubrowski A. Comparing self-guided learning and educator-guided learning formats for simulation-based clinical training. Journal of Advanced Nursing. 2010;66(8):1832–44.
10. Brydges R, Peets A, Issenberg SB, Regehr G. Divergence in student and educator conceptual structures during auscultation training. Medical Education. 2013;47(2):198–209.
11. Buesing J, Weng Y, Kugler J, Wang L, Blaha O, Hom J, et al. Handheld Ultrasound Device Usage and Image Acquisition Ability Among Internal Medicine Trainees: A Randomized Trial. Journal of graduate medical education. 2021;13(1):76–82.
12. Chandelkar UK, Rataboli PV, Kulkarni MS. Assessment of impact of small group teaching over didactic lectures and self-directed learning among second year bds students in general and dental pharmacology in goa medical college. Pharmacologyonline. 2014;3:51–7.
13. Chen LX, Fuller T, McLntire DD, Kho KA. Introduction to Open Surgical Skills Curriculum: Randomized Trial of Self-Paced vs Group Video Tutorial Viewing. Journal of Surgical Education. 2019;76(2):453–8.
14. Crihfield EG, Uppalapati P, Abittan B, Laibangyang A, Brahmbhatt S, Burlingame M, et al. Development of laparoscopic skills in skills-naïve trainees using self-directed learning with take-home laparoscopic trainer boxes. Surgery Open Science. 2023;16:82–93.
15. Denadai R, Toledo AP, Oshiiwa M, Saad-Hossne R. Acquisition of suture skills during medical graduation by instructor-directed training: A randomized controlled study comparing senior medical students and faculty surgeons. Updates in Surgery. 2013;65(2):131–40.
16. Dutra B, Lissauer M, Rashid H. Nutrition Education on the Wards: A Self-Study Module for Improving Medical Student Knowledge of Nutrition Assessment and Interventions. MedEdPORTAL : the journal of teaching and learning resources. 2020;16:10968.
17. Ellington DR, Shum PC, Dennis EA, Willis HL, Szychowski JM, Richter HE. Female Pelvic Floor Immersive Simulation: A Randomized Trial to Test the Effectiveness of a Virtual Reality Anatomic Model on Resident Knowledge of Female Pelvic Anatomy. Journal of Minimally Invasive Gynecology. 2019;26(5):897–901.
18. Gao F, Li J, Xu J, Jia S, Xu L, Li S, et al. Evaluating the appropriateness of AccessMedicine in integrated biochemistry learning for chinese medical students.

Biochemistry and molecular biology education : a bimonthly publication of the International Union of Biochemistry and Molecular Biology. 2019;47(3):272–8.
19. Gawad N, Zevin B, Bonrath EM, Dedy NJ, Louridas M, Grantcharov TP. Introduction of a comprehensive training curriculum in laparoscopic surgery for medical students: A randomized trial. Surgery (United States). 2014;156(3):698–706.
20. Giglioli S, Boet S, De Gaudio AR, Linden M, Schaeffer R, Bould MD, et al. Self-directed deliberate practice with virtual fiberoptic intubation improves initial skills for anesthesia residents. Minerva anestesiologica. 2012;78(4):456–61.
21. Haq R, Li B, Jovicic A, Dastur D, Trinkaus M, Kong A. Web-based Oncology Educational Tool for Medical Trainees on Oncology Rotation-Results of a Pilot Study. Journal of cancer education : the official journal of the American Association for Cancer Education. 2018;33(4):788–97.
22. Holm E, Holte M, Pedersen H, Hansen PD. Teaching geriatrics using evidence based educational methods-a Danish case study. European Geriatric Medicine. 2013;4(2):95–8.
23. Hossain MS, Shofiqul Islam M, Glinsky JV, Lowe R, Lowe T, Harvey LA. A massive open online course (MOOC) can be used to teach physiotherapy students about spinal cord injuries: a randomised trial. Journal of physiotherapy. 2015;61(1):21–7.
24. Jittitaworn W, Wisanskoonwong P. Self-directed learning about normal birth with web-based support for nurse-midwife students in Thailand: A mixed methods study. Midwifery. 2023;126:103813.
25. Junhasavasdikul D, Sukhato K, Srisangkaew S, Theera-Ampornpunt N, Anothaisintawee T, Dellow A. Cartoon versus traditional self-study handouts for medical students: CARTOON randomized controlled trial. Medical Teacher. 2017;39(8):836–43.
26. Kameda T, Koibuchi H, Konno K, Taniguchi N. Self-learning followed by telepresence instruction of focused cardiac ultrasound with a handheld device for medical students: a preliminary study. Journal of Medical Ultrasonics. 2022;49(3):415–23.
27. Khan T, Cinnor B, Gupta N, Hosford L, Bansal A, Olyaee MS, et al. Didactic training vscomputer-based self-learning in the prediction of diminutive colon polyp histology by trainees: A randomized controlled study. Endoscopy. 2017;49(12):1243–50.
28. Kirkpatrick JS. A comparison C1-C2 transarticular screw placement after self-education and mentored education of orthopaedic residents. Journal of Spinal Disorders and Techniques. 2012;25(6):E155–60.
29. Kooloos JG, de Waal Malefijt MC, Ruiter DJ, Vorstenbosch MA. Loosely-guided, self-directed learning versus strictly-guided, station-based learning in gross anatomy laboratory sessions. Anatomical Sciences Education. 2012;5(6):340–6.
30. Kulier R, Gülmezoglu AM, Zamora J, Plana MN, Carroli G, Cecatti JG, et al. Effectiveness of a clinically integrated e-learning course in evidence-based medicine for reproductive health training: A randomized trial. JAMA. 2012;308(21):2218–25.
31. Le CK, Lewis J, Steinmetz P, Dyachenko A, Oleskevich S. The Use of Ultrasound Simulators to Strengthen Scanning Skills in Medical Students: A Randomized Controlled Trial. JOURNAL OF ULTRASOUND IN MEDICINE. 2019;38(5):1249–57.
32. Leavitt RM, Arpin PA, Nielsen BM, Mason NL. Independent learning of the sonographic FAST exam technique using a tablet-based training module. American journal of disaster medicine. 2021;16(2):95–104.

33. Lee GI, Lee MR. Can a virtual reality surgical simulation training provide a self-driven and mentor-free skills learning? Investigation of the practical influence of the performance metrics from the virtual reality robotic surgery simulator on the skill learning and associated cognitive workloads. Surgical Endoscopy. 2018;32(1):62–72.
34. LeFlore JL, Anderson M. Alternative Educational Models for Interdisciplinary Student Teams. SIMULATION IN HEALTHCARE-JOURNAL OF THE SOCIETY FOR SIMULATION IN HEALTHCARE. 2009;4(3):135–42.
35. Lian A, Rippey JCR, Carr PJ. Teaching medical students ultrasound-guided vascular access - which learning method is best? Journal of Vascular Access. 2017;18(3):255–8.
36. Love T, Wiese LAK, Duncan V, Bertrand H. Does self-directed learning address gaps in nursing student knowledge of Alzheimer's disease? EDUCATIONAL GERONTOLOGY. 2023;49(8):673–86.
37. MacArthur-Beadle I, Nair DVK, Cook NJ, Yi M, Jones R, Beasley SW, et al. Master and Apprentice or a Slave to Technology? A Randomized Controlled Trial of Minimal Access Surgery Simulation-Based Training Techniques. Journal of Laparoendoscopic and Advanced Surgical Techniques. 2020;30(12):1263–71.
38. MacDonell-Yilmaz R, Murillo A, Welch J. PEDIATRIC OPIOID ANALGESIA SELF-INSTRUCTION SYSTEM (PEDOASIS): AN EFFECTIVE EDUCATION TOOL. Pediatric Blood and Cancer. 2022;69(SUPPL 2):S115–6.
39. Mahler SA, Wolcott CJ, Swoboda TK, Wang H, Arnold TC. Techniques for teaching electrocardiogram interpretation: self-directed learning is less effective than a workshop or lecture. Medical Education. 2011;45(4):347–53.
40. Marei HF, Donkers J, Van Merrienboer JJG. The effectiveness of integration of virtual patients in a collaborative learning activity. Medical Teacher. 2018;40:S96–103.
41. Margaret Shanthi FX, Blessed Winston A, Kumar A, Das S, Sen S, Ranjalkar J, et al. Evaluation of efficacy of tutorials in pharmacology for 2nd year undergraduate medical students in India. Research Journal of Pharmacy and Technology. 2016;9(2):153–6.
42. Mayer-Hirshfeld I, Agarwal GG, Jackson JS. SVI Self-Study Guide: A Study Tool for Independent Preparation for the AAMC Standardized Video Interview. MedEdPORTAL : the journal of teaching and learning resources. 2019;15:10805.
43. McCarthy N, Neville K, Pope A, Barry L, Livingstone V. Effectiveness of a proficiency-based progression e-learning approach to training in communication in the context of clinically deteriorating patients: a multi-arm randomised controlled trial. BMJ Open [Internet]. 2023;13(8).
44. Mpotos N, Yde L, Calle P, Deschepper E, Valcke M, Peersman W, et al. Retraining basic life support skills using video, voice feedback or both: A randomised controlled trial. Resuscitation. 2013;84(1):72–7.
45. Nathan A, Patel S, Georgi M, Fricker M, Asif A, Ng A, et al. Virtual classroom proficiency-based progression for robotic surgery training (VROBOT): a randomised, prospective, cross-over, effectiveness study. Journal of Robotic Surgery. 2023;17(2):629–35.
46. Noh GO, Kim DH. Effectiveness of a self-directed learning program using blended coaching among nursing students in clinical practice: a quasi-experimental research design. BMC medical education. 2019;19(1):225.

47. Park JY, Woo CH, Yoo JY. Effects of Blended Cardiopulmonary Resuscitation and Defibrillation E-learning on Nursing Students' Self-efficacy, Problem Solving, and Psychomotor Skills. CIN-COMPUTERS INFORMATICS NURSING. 2016;34(6):272–80.
48. Pedersen TH, Kasper N, Roman H, Egloff M, Marx D, Abegglen S, et al. Self-learning basic life support: A randomised controlled trial on learning conditions. Resuscitation. 2018;126:147–53.
49. Pioche M, Rivory J, Nishizawa T, Uraoka T, Touzet S, O'Brien M, et al. Randomized comparative evaluation of endoscopic submucosal dissection self-learning software in France and Japan. Endoscopy. 2016;48(12):1076–83.
50. Ramar K, De Moraes AG, Selim B, Holets S, Oeckler R. Effectiveness of hands-on tutoring and guided self-directed learning versus self-directed learning alone to educate critical care fellows on mechanical ventilation - a pilot project. Medical education online. 2016;21:32727.
51. Raupach T, Harendza S, Anders S, Schuelper N, Brown J. How can we improve teaching of ECG interpretation skills? Findings from a prospective randomised trial. Journal of Electrocardiology. 2016;49(1):7–12.
52. Rong Y, Glozier N, Luscombe GM, Davenport TA, Huang Y, Hickie IB. Improving Knowledge and Attitudes towards Depression: A controlled trial among Chinese medical students. BMC Psychiatry [Internet]. 2011;11.
53. Ryznar E, Hamaoka D, Lloyd RB. Pilot Study of an Online Self-Directed Learning Module for Medical Decision-Making Capacity. Academic psychiatry : the journal of the American Association of Directors of Psychiatric Residency Training and the Association for Academic Psychiatry. 2020;44(4):408–12.
54. Rzouq F, Vennalaganti P, Pakseresht K, Kanakadandi V, Parasa S, Mathur SC, et al. In-class didactic versus self-directed teaching of the probe-based confocal laser endomicroscopy (pCLE) criteria for Barrett's esophagus. Endoscopy. 2016;48(2):123–7.
55. Saiboon IM, Musni N, Daud N, Shamsuddin NS, Jaafar MJ, Hamzah FA, et al. Effectiveness of Self-Directed Small-Group-Learning Against Self-Directed Individual-Learning Using Self-Instructional-Video in Performing Critical Emergency Procedures Among Medical Students in Malaysia: A Single-Blinded Randomized Controlled Study. Clinical Simulation in Nursing. 2021;56:46–56.
56. Sakamoto Y, Okamoto S, Shimizu K, Araki Y, Hirakawa A, Wakabayashi T. Comparative Prospective Study of Microvascular Anastomosis Training by Self-Learning or with Expert Instruction. World Neurosurgery. 2018;118:e818–24.
57. Schmidt MW, Kowalewski KF, Trent SM, Benner L, Mueller-Stich BP, Nickel F. Self-directed training with e-learning using the first-person perspective for laparoscopic suturing and knot tying: a randomised controlled trial Learning from the surgeon's real perspective. Surgical Endoscopy and Other Interventional Techniques. 2020;34(2):869–79.
58. Shah R, Sibbald M, Jaffer N, Probyn L, Cavalcanti RB. Online self-study of chest X-rays shows no difference between blocked and mixed practice. Medical Education. 2016;50(5):540–9.
59. Sheakley ML, Bauler TJ, Vandre DD, Woodwyk A, Dickinson BL. Effectiveness of instructor-guided independent learning in comparison to traditional didactic lecture in the preclinical medical curriculum: A retrospective cohort study. Medical Teacher. 2019;41(7):795–801.

60. Shenoy PJ, Rao RR. Crossword puzzles versus student-led objective tutorials (SLOT) as innovative pedagogies in undergraduate medical education. Scientia Medica. 2021;31(1):1–7.
61. Smith SCL, Saltzman J, Shivaji UN, Lethebe BC, Cannatelli R, Ghosh S, et al. Randomized controlled study of the prediction of diminutive/small colorectal polyp histology using didactic versus computer-based self-learning module in gastroenterology trainees. Digestive Endoscopy. 2019;31(5):535–43.
62. Snyder CW, Vandromme MJ, Tyra SL, Hawn MT. Proficiency-based laparoscopic and endoscopic training with virtual reality simulators: a comparison of proctored and independent approaches. Journal of Surgical Education. 2009;66(4):201–7.
63. Snyder CW, Vandromme MJ, Tyra SL, Hawn MT. Retention of colonoscopy skills after virtual reality simulator training by independent and proctored methods. The American surgeon. 2010;76(7):743–6.
64. Sox CM, Tenney-Soeiro R, Lewin LO, Ronan J, Brown M, King M, et al. Efficacy of a Web-Based Oral Case Presentation Instruction Module: Multicenter Randomized Controlled Trial. Academic Pediatrics. 2018;18(5):535–41.
65. Tam M, Hart AR, Williams SM, Holland R, Heylings D, Leinster S. Evaluation of a computer program ('disect') to consolidate anatomy knowledge: A randomised-controlled trial. Medical Teacher. 2010;32(3):E138–42.
66. Tao Y, Li L, Xu Q, Jiang A. Development of a nursing education program for improving Chinese undergraduates' self-directed learning: A mixed-method study. Nurse Education Today. 2015;35(11):1119–24.
67. Thalanjeri P, Balakrishnan G, Kalpana B, Ali SI. Impact of Autonomous Learning for Advanced Acquisition of Physiology (ALAAP) module among undergraduate health-care professional students. Indian Journal of Physiology and Pharmacology. 2022;66(2):146–52.
68. Thompson M, Johansen D, Stoner R, Jarstad A, Sorrells R, McCarroll ML, et al. Comparative effectiveness of a mnemonic-use approach vs. self-study to interpret a lateral chest X-ray. Advances in physiology education. 2017;41(4):518–21.
69. Tiu J, Cheng EX, Hung TC, Yu CC, Lin T, Schwass D, et al. Effectiveness of Crown Preparation Assessment Software As an Educational Tool in Simulation Clinic: A Pilot Study. Journal of Dental Education. 2016;80(8):1004–11.
70. Toumas M, Basheti IA, Bosnic-Anticevich SZ. Comparison of small-group training with self-directed internet-based training in inhaler techniques. American Journal of Pharmaceutical Education [Internet]. 2009;73(5).
71. Uhm TH, Kim JH. Effectiveness of 5, 10, 15-min video self-instruction in cardiopulmonary resuscitation training. Research Journal of Pharmacy and Technology. 2018;11(2):649–52.
72. Wilson E, Janssens S, McLindon LA, Hewett DG, Jolly B, Beckmann M. Improved laparoscopic skills in gynaecology trainees following a simulation-training program using take-home box trainers. Australian & New Zealand Journal of Obstetrics & Gynaecology. 2019;59(1):110–6.
73. Xiong P, Zhang J, Wang X, Wu TL, Hall BJ. Effects of a mixed media education intervention program on increasing knowledge, attitude, and compliance with standard precautions among nursing students: A randomized controlled trial. American Journal of Infection Control. 2017;45(4):389–95.

74. Zayed MA, Lilo EA, Lee JT. Impact of an Interactive Vascular Surgery Web-Based Educational Curriculum on Surgical Trainee Knowledge and Interest. Journal of Surgical Education. 2017;74(2):251–7.
75. Zhang Y, Simler MAZ, Stabile I. Supported self-directed learning of Clinical Anatomy: A pilot study of doughnut rounds. European Journal of Anatomy. 2017;21(4):319–24.
76. Zhao YC, Kennedy G, Yukawa K, Pyman B, O'Leary S. Improving temporal bone dissection using self-directed virtual reality simulation: Results of a randomized blinded control trial. Otolaryngology - Head and Neck Surgery. 2011;144(3):357–64.
77. Zundel S, Blumenstock G, Herrmann-Werner A, Trueck M, Schmidt A, Wiechers S. Undescended testis? How best to teach the physical examination. Journal of Pediatric Urology. 2016;12(6):406.e1-406.e6.

Search Strategy

August 12, 2009 - October 11, 2023
(date range for all results unless otherwise specified)

Cinahl
Embase
OVID Medline
PsychINFO
Web of Science

Database	Search parameters
CINAHL (n=291)*<br><br>* Only allows date to be by month - August was selected	1. (MH "Self Directed Learning+")<br>2. ((self-directed or independent or self or individualized or individual) W0 (study or studies or studying or learn* or instruction or instructing or educat*)) NOT ‘individual studies’<br>3. “inquiry method” OR “independent learning” OR “autonomous learning” OR Heutagogy OR “self assessment in learning” OR “self initiative in learning” OR “learner autonomy”<br>4. Self W0 (education OR instruction OR study OR teaching)<br>5. Self W0 (determined OR driven OR initiated OR managed OR motivated OR organized OR planned OR scheduled) W0 learning<br>6. Learner W0 (centered OR driven OR initiated) W0 education<br>7. 1 or 2 or 3 or 4 or 5 or 6<br>8. (MH "Specialties, Medical+/ED")<br>9. (MH "Health Occupations+/ED")<br>10. (MH "Education, Medical+")<br>11. (MH "Health Personnel+")<br>12. 8 or 9 or 10 or 11<br>13. 7 and 12<br>14. (MH "Comparative Studies+")<br>15. Compar*<br>16. (controlled W0 (study or studies or trial or trials))<br>17. Cohort<br>18. 14 or 15 or 16 or 17<br>19. 13 and 18
Embase (n=3767)	1. 'self-directed learning'/exp<br>2. ((self-directed or independent or self or individualized or individual) next/1 (study or studies or studying or learn* or instruction or instructing or educat*))<br>3. “inquiry method” OR “independent learning” OR “autonomous learning” OR Heutagogy OR “self assessment in learning” OR “self initiative in learning” OR “learner autonomy”<br>4. Self next/1 (education OR instruction OR study OR teaching)<br>5. Self next/1 (determined OR driven OR initiated OR managed OR motivated OR organized OR planned OR scheduled) next/1 learning<br>6. Learner next/1 (centered OR driven OR initiated) next/1 education<br>7. 1 or 2 or 3 or 4 or 5 or 6<br>8. 'medicine'/exp

	9. 'medical profession'/exp<br>10. 'medical education'/exp<br>11. 'health care personnel'/exp<br>12. 8 or 9 or 10 or 11<br>13. 7 and 12<br>14. 'comparative study'/exp<br>15. compar*<br>16. (controlled next/1 (study or studies or trial or trials))<br>17. Cohort<br>18. 14 or 15 or 16 or 17<br>19. 13 and 18
OVID Medline (n=1397)	1. exp Self-Directed Learning as Topic/<br>2. (((self-directed or independent or self or individualized or individual) adj (study or studies or studying or learn$ or instruction or instructing or educat$)).mp) NOT ‘individual studies’.mp<br>3. “inquiry method” OR “independent learning” OR “autonomous learning” OR Heutagogy OR “self assessment in learning” OR “self initiative in learning” OR “learner autonomy”<br>4. Self adj (education OR instruction OR study OR teaching)<br>5. Self adj (determined OR driven OR initiated OR managed OR motivated OR organized OR planned OR scheduled) adj learning<br>6. Learner adj (centered OR driven OR initiated) adj education<br>7. 1 or 2 or 3 or 4 or 5 or 6<br>8. exp Specialties, Medical/ed<br>9. exp Health Occupations/ed<br>10. exp Education, Medical/<br>11. exp Health Personnel/<br>12. 8 or 9 or 10 or 11<br>13. 7 and 12<br>14. exp Comparative Study/<br>15. Compar$<br>16. (controlled adj (study or studies or trial or trials)).mp<br>17. Cohort.mp<br>18. 14 or 15 or 16 or 17<br>19. 13 and 18
PsycINFO via Ebsco (n=61)*<br><br>* Only allows date to be by month - August was selected	1. (MA "Self Directed Learning+")<br>2. ((self-directed or independent or self or individualized or individual) W0 (study or studies or studying or learn* or instruction or instructing or educat*)) NOT ‘individual studies’<br>3. “inquiry method” OR “independent learning” OR “autonomous learning” OR Heutagogy OR “self assessment in learning” OR “self initiative in learning” OR “learner autonomy”<br>4. Self W0 (education OR instruction OR study OR teaching)<br>5. Self W0 (determined OR driven OR initiated OR managed OR motivated OR organized OR planned OR scheduled) W0 learning<br>6. Learner W0 (centered OR driven OR initiated) W0 education<br>7. 1 or 2 or 3 or 4 or 5 or 6<br>8. MA Specialties, Medical<br>9. MA Health Occupations<br>10. MA Education, Medical<br>11. MA Health Personnel<br>12. 8 or 9 or 10 or 11

	13. 7 and 12<br>14. MA Comparative Study<br>15. Compar*<br>16. (controlled W0 (study or studies or trial or trials))<br>17. Cohort<br>18. 14 or 15 or 16 or 17<br>19. 13 and 18
Web of Science<br>(n=2814)	1. ALL=('self-directed learning')<br>2. (TI=((self-directed or independent or self or individualized or individual) near/0 (study or studies or studying or learn* or instruction or instructing or educat*))) OR (AB=((self-directed or independent or self or individualized or individual) near/0 (study or studies or studying or learn* or instruction or instructing or educat*)))<br>3. ALL=("inquiry method" OR "independent learning" OR "autonomous learning" OR Heutagogy OR "self assessment in learning" OR "self initiative in learning" OR "learner autonomy")<br>4. (TI=(Self near/0 (education OR instruction OR study OR teaching))) OR (AB=(Self near/0 (education OR instruction OR study OR teaching)))<br>5. (TI=(Self near/0 (determined OR driven OR initiated OR managed OR motivated OR organized OR planned OR scheduled) near/0 learning)) OR (AB= (Self near/0 (determined OR driven OR initiated OR managed OR motivated OR organized OR planned OR scheduled) near/0 learning))<br>6. (TI=(Learner near/0 (centered OR driven OR initiated) near/0 education)) OR (AB=(Learner near/0 (centered OR driven OR initiated) near/0 education))<br>7. 1 or 2 or 3 or 4 or 5 or 6<br>8. ALL=(therapist* or doctor* or physician* or "medical student*" or resident* or nurse* or nursing or dietician* or "physical therapist*" or dietetic* or "medical education" or "medical teaching" or dentist* or dental or hygienist* or psychologist* or caregiver* or orthodondist* or periodontist* or prosthodontist* or pharmacist* or audiologist* or audiometrist* or "hearing therapist*" or "occupational therapy" or optometrist* or "oral hygienist" or "orthodontic technician*" or paramedic* or "physicians assistant*")<br>9. ALL=(prophylactician* or "speech language pathologist*" or "speech pathologist*" or "speech therapist*" or "x-ray technologist*" or "x-ray w technician*" or hospitalist* or surgeon* or anesthesist* or anesthesiologist* or cardiologist* or "general practitioner*" or gerontologist* or nephrologist* or pathologist* or pediatrician or podiatrist* or psychiatrist* or urologist* or allergist* or immunologist* or dermatologist* or "family practitioner*" or medicinist* or neurologist* or gynecologist* or obstetrician* or venereologist*)<br>10. 8 or 9<br>11. 7 and 10<br>12. ALL='comparative study'<br>13. compar*<br>14. (TI=(controlled near/0 (study or studies or trial or trials))) OR (AB=(controlled near/0 (study or studies or trial or trials)))<br>15. ALL=Cohort<br>16. 12 or 13 or 14 or 15<br>17. 11 and 16<br>18. DOP=(2009-08-12/2023-10-11) AND #17
